# Small-Cell Lung Carcinoma Associated With Cystic Airspaces: A Case Report

**DOI:** 10.1155/carm/5314337

**Published:** 2025-07-21

**Authors:** Hirokazu Touge, Haruki Masui, Mitsuhiro Yamamoto, Tomoyuki Ikeuchi, Tomohiro Sakamoto, Katsuyuki Tomita, Akira Yamasaki

**Affiliations:** ^1^Department of Respiratory Medicine, NHO Yonago Medical Center, 4-17-1 Kuzumo, Yonago 683-0006, Tottori, Japan; ^2^Center for Clinical Residency Program, NHO Yonago Medical Center, 4-17-1 Kuzumo, Yonago 683-0006, Tottori, Japan; ^3^Division of Respiratory Medicine and Rheumatology, Department of Multidisciplinary Internal Medicine, School of Medicine, Faculty of Medicine, Tottori University, 36-1 Nishimachi, Yonago 683-8504, Tottori, Japan

**Keywords:** cyst, mimicking pneumonia, small-cell lung carcinoma

## Abstract

Small-cell lung carcinoma (SCLC) associated with cystic airspaces is rare. We describe the case of a 68-year-old man who was referred to our hospital because of a cystic lesion detected on chest radiography. Initial computed tomography revealed a small nodule abutting the cystic airspace due to paraseptal emphysema in the right lower lobe. Histopathological examination of lymphadenopathy indicated SCLC. Postchemotherapy, recurrence appeared as a thick-walled cystic airspace with an exophytic nodule along the cyst wall, mimicking pneumonia. Additional chemotherapy, but not antibiotic therapy, led to a reduction in the wall thickness and nodules. This case emphasizes unresponsiveness to antibiotic therapy, especially in patients with risk factors, highlighting the diagnostic pitfall that may delay timely cancer treatment.

## 1. Introduction

Several rare malignant lung lesions that are often misdiagnosed include lung cancer (LC) associated with cystic airspaces. Computed tomography (CT) is frequently used for LC screening and follow-up. LC associated with cystic airspaces is often missed or misinterpreted because of its unique visualization, which can overlap with benign entities, such as infections. According to several studies, the incidence of these rare lesions is 0.5%–3.7% [1–5]. Most studies have reported that adenocarcinoma is the predominant histological type of LC associated with cystic airspaces, with a systematic review and meta-analysis reporting an incidence of up to 88% [6]. Squamous cell carcinoma is the second most prevalent cancer. Here, we describe an extremely rare case of small-cell lung carcinoma (SCLC) associated with a cystic airspace with a thickened wall and an adjacent nodule that progressed postrecurrence, mimicking pneumonia.

## 2. Case Presentation

**Figure 1:**
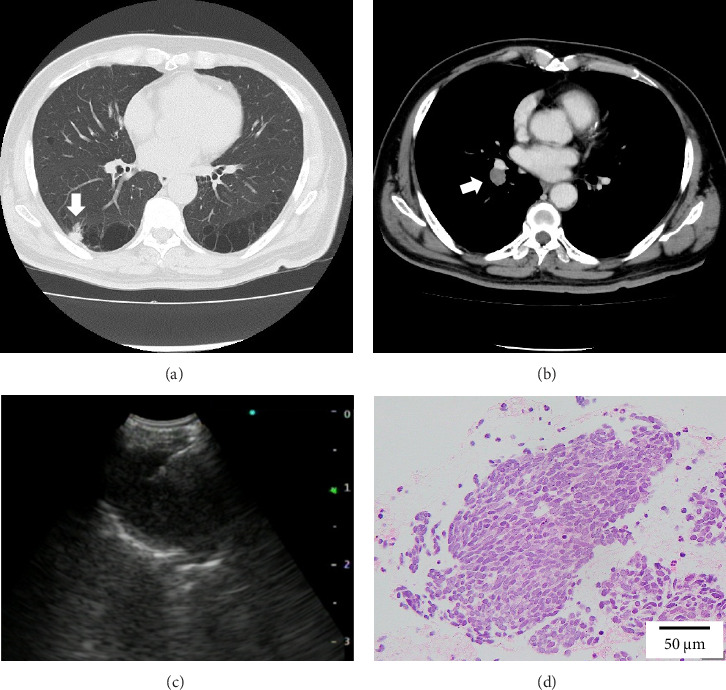
Axial computed tomography (CT) scan revealing a solid nodule (arrow) 8 mm in diameter abutting the cranial portion of 6 cm thin-walled cystic airspace in the right lower lobe (a); contrast-enhanced CT showing the presence of enlarged interlobar lymph node (arrow) in Station 11B (b); image of endobronchial ultrasound-guided transbronchial needle aspiration (EBUS-TBNA) showing homogenous echogenic interlobar lymph node (Station 11B) measuring 1.5 cm (c); pathological findings of biopsy obtained from EBUS-TBNA with a hematoxylin and eosin stain (d). A tumor composed of nests of small cells with fine granular salt and pepper chromatin and scarce cytoplasm (H&E stain, × 100).

A 68-year-old male smoker underwent a periodic checkup for LC and had a normal chest radiograph obtained 30 months before presentation. The patient presented at our hospital with a thin-walled cyst in the right lower lobe. His medical history of 2 years included significant complaints of exertional dyspnea for chronic obstructive pulmonary disease with Global Initiative for Chronic Obstructive Lung Disease Stage II. Regular medications included long-acting muscarinic antagonists. The patient was a former office worker with no prior exposure to asbestos. CT revealed hilar lymphadenopathy with a solid nodule 8 mm in diameter abutting the cranial portion of a 6 cm thin-walled cystic airspace in the right lower lobe (Figures 1(a) and 1(b)). Progastrin-releasing peptide (Pro-GRP) levels in the plasma had increased and exceeded the reference value of 193 pg/mL (normal range: 0–81 pg/mL). Endobronchial ultrasound-guided transbronchial needle aspiration of the right interlobar lymph node (Station 11 B) revealed SCLC composed of nests of small cells with finely granular salt, pepper chromatin, and scarce cytoplasm (Figures 1(c) and 1(d)). Bone scintigraphy revealed no bone metastasis. Neither enhanced brain magnetic resonance imaging nor abdominal CT showed any evidence of metastases. The patient was diagnosed with Stage IIB SCLC at clinical stage T1bN1M0. He underwent four cycles of concurrent chemoradiotherapy with etoposide (100 g/m^2^) and cisplatin (80 mg/m^2^) as first-line treatment. There are no indications of metastasis or recurrence during routine check-ups every 3 months.

**Figure 2:**
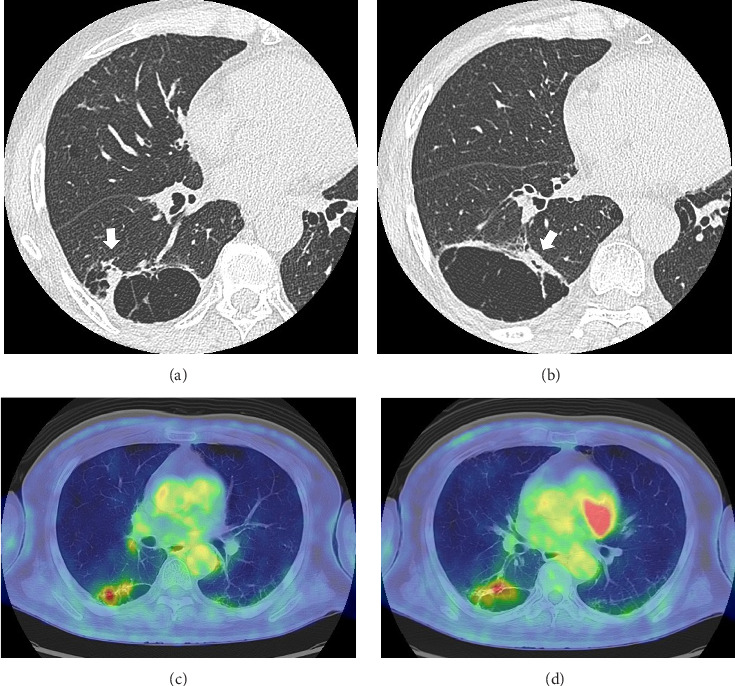
High-resolution computed tomography (HRCT) (a and b), and 18F-fluorodeoxyglucose positron emission tomography/CT (c and d) images 14 months after initial chemotherapy showing disseminated thickened-walled cystic airspaces and two enlarged nodules (arrows) measuring 1.2 cm in the right lower lobe with a high 18F-FDG uptake.

**Figure 3:**
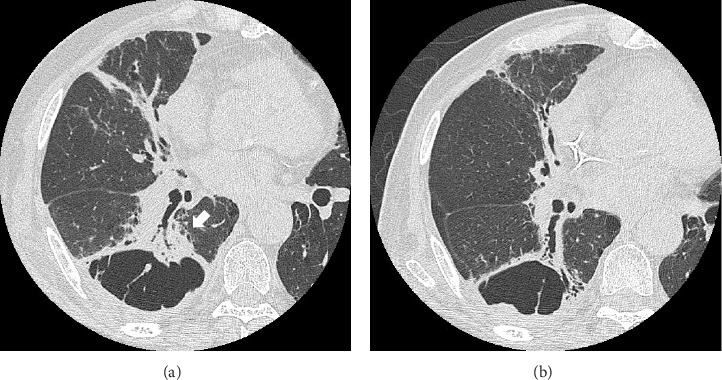
High-resolution computed tomography (HRCT) reveals (a) a thick-walled cystic space with an exophytic enlarged solid nodule (arrow) along the cyst wall and a new shadow of pseudotumor mimicking consolidation in the right lower lobe during third-line chemotherapy; (b) HRCT showing the diminished nodule adjacent to the thickened cystic airspace wall and the cysts fusing into bizarre shapes after 4 months of starting fourth-line therapy.

Fourteen months after first-line chemotherapy, CT showed an irregular thick-walled cystic airspace and two enlarged nodules measuring 1.2 cm (Figures 2(a) and 2(b)). 18F-fluorodeoxyglucose positron emission tomography/CT (18F-FDG-PET) revealed homogenous uptake in a solid tumor (standard uptake value max: 5.6) with circumferential wall thickening in the right lower lobe, suggestive of a progression of the initial disease, as well as the regression of the right hilar lymphadenopathy (Figures 2(c) and 2(d)). Four cycles of carboplatin (area under the curve = 5), etoposide (100 mg/m^2^), and durvalumab (1500 mg/body) were administered to treat recurrent SCLC. After second-line chemotherapy, durvalumab (1500 mg/body) was administered as an outpatient treatment. After two third-line cycles, the patient was admitted to our hospital 1 week after experiencing right-sided chest pain and coughing. Upon admission, the examination revealed a body temperature of 36.8°C, blood pressure of 118/84 mmHg, respiratory rate of 18 times per minute, and heart rate of 97 beats per minute. Respiratory auscultation revealed decreased breathing capacity. The oxygen saturation was 96% in ambient air. Laboratory examinations conducted at our hospital revealed a white blood cell (WBC) count of 19.4 × 10^9^/L and a C-reactive protein (CRP) level of 23.02 mg/L (normal: < 5 mg/L). Sputum cultures indicated infection with *Enterococcus faecalis* and *Klebsiella pneumoniae*. The plasma pro-GRP level was elevated to 621 pg/mL. CT revealed an enlarged solid nodule adjusted to the cystic airspaces, and a new appearance of consolidation localized to the irradiated field, and intracystic fluid in the right lower lobe without any lymphadenopathy, which was suspected to be radiation-induced pneumonitis or pneumonia (Figure 3(a)). Two weeks after starting broad-spectrum antibiotics, including piperacillin–tazobactam, he developed symptoms of right chest pain and cough and showed a decline in infection-related marker values, with a WBC count of 15.2 × 10^9^/L and CRP level of 7.46 mg/L. Cytological specimens obtained via bronchial washing and brushing confirmed the diagnosis of SCLC. Fourth-line therapy with amrubicin (40 mg/m^2^) rapidly resulted in consolidation and wall thickening in the right lower lobe (Figure 3(b)), with almost normal infection-related marker values: WBC count of 12.2 × 10^9^/L and CRP level of 1.68 mg/L. The patient remained alive without disease progression at the 6-month follow-up, with a CT scan every 3 months.

## 3. Discussion

Solitary nodules or multiple nodules are the most common radiological manifestations of LC. This report describes an extremely rare case of SCLC presenting as a nodule with cystic airspaces. A review of the literature reveals that there has only been one previous report of SCLC associated with cystic airspaces [6]. The term “cystic” has recently been redefined as a descriptor of lesions characterized by central air-equivalent attenuation but not by any etiology, including emphysema [7]. Our patient presented with preexisting paraseptal emphysema and tumor growth along the walls of the cystic airspaces. Recurrence on CT manifested as a thick-walled cystic airspace with an exophytic solid nodule along the cyst wall, mimicking pneumonia.

A systematic review and meta-analysis indicate that adenocarcinomas account for most cases of LC associated with cystic airspaces. In addition, there have been reports on squamous cell carcinomas and, to a lesser extent, large-cell carcinomas [8]. LC associated with cystic airspaces has been recognized as a cause of missed cancer in LC screening trials [9, 10]. In addition, a rapid growth of the peripheral type of SCLC [11] makes it difficult to obtain early phase images, which explains why SCLC associated with cystic airspace is so uncommon.

Based on the location and the morphology of the solid components in relation to the cystic airspaces, previous studies have proposed categorizing the morphological types of LC linked to cystic airspaces [4, 12]. Types I and II involve cystic airspaces with solid exophytic and endophytic nodules. Cystic airway involvement with asymmetric or circumferential wall thickening was categorized as Type III. On the other hand, a multilocular cystic lesion with inserted solid tissue was defined as Type IV. In the present case, pretreatment CT revealed a small nodule abutting the external aspect of the lung cyst, which was classified as Type I. During the second recurrence, CT revealed irregular circumferential thickening of the cyst wall and a multicystic lesion containing an area of soft tissue attenuation; thus, it was classified as Type III or Type IV.

Unfortunately, because of its uncommon morphological presentation, LC associated with cystic airspaces often remains unrecognized, thereby increasing the risk of delayed diagnosis [13, 14]. 18F-FDG-PET-CT imaging may help confirm suspected LC; however, the diagnostic ability of this analysis is limited to LC adjoining the cystic airspaces [15]. Types III and IV LC associated with cystic airspaces are challenging to distinguish from infections, such as pneumonia and inflammatory cysts, even with 18F-FDG-PET-CT.

Immunotherapy with durvalumab as a consolidation treatment after concurrent platinum-based chemoradiotherapy has helped to control SCLC recurrence, resulting in longer relapse-free periods. However, our patient experienced a recurrence after two cycles of durvalumab. In our case, the size of the cystic airspaces remained unchanged, although the cystic wall thickened upon follow-up high-resolution CT. In particular, the cystic wall partially increased in thickness, adjusting for the nodule and consolidation. As a result of the chemotherapy, the partially thickened cystic wall became thinner. The cystic appearance and growth along the wall of a preexisting cystic airspace due to paraseptal emphysema were altered by chemotherapy.

In SCLC, submucosal invasion occurs locally, followed by the invasion of the peribronchial connective tissue. Epithelial–mesenchymal transition (EMT) is an evolutionarily conserved mechanism that can enhance mobility and resistance to cell death and is the driving force of tumor cell invasion [16, 17]. Our case was assumed to be atypical paraseptal emphysema without upper-lobe predominance or adjacent centrilobular emphysema. Since airway EMT activity is high in patients with emphysema [18, 19], in our case, the SCLC extended along the bronchovascular bundle, which was tethered to the surface of the adjacent cystic airspaces, resulting in a consolidation-like appearance on CT.

## 4. Conclusion

SCLC associated with cystic airspaces is rare and can be misdiagnosed as pneumonia due to its curious extended pattern. This case emphasizes the importance of considering malignancies in patients with cystic lung lesions that are unresponsive to antibiotic therapy, particularly in patients with risk factors. This highlights diagnostic pitfalls that may delay timely cancer treatment.

## Data Availability

The data that support the findings of this study are available on request from the corresponding author. The data are not publicly available due to privacy or ethical restrictions.
